# Maternal Obesity in Mice Exacerbates the Allergic Inflammatory Response in the Airways of Male Offspring

**DOI:** 10.3390/nu11122902

**Published:** 2019-12-01

**Authors:** Rodrigo Rodrigues e-Lacerda, Caio Jordão Teixeira, Silvana Bordin, Edson Antunes, Gabriel Forato Anhê

**Affiliations:** 1 Department of Pharmacology, Faculty of Medical Sciences, State University of Campinas, 13083-881 Campinas, SP, Brazilcaiojteixeira@gmail.com (C.J.T.); edson.antunes@uol.com.br (E.A.); 2 Department of Physiology and Biophysics, Institute of Biomedical Sciences, University of Sao Paulo, 05508-900 Sao Paulo, SP, Brazil; sbordin@icb.usp.br

**Keywords:** obesity, pregnancy, allergic airway disease, offspring, high fat diet

## Abstract

It was previously demonstrated that non-allergen-sensitized rodents born to mothers exposed to a high-fat diet (HFD) spontaneously develop lower respiratory compliance and higher respiratory resistance. In the present study, we sought to determine if mice born to mothers consuming HFD would exhibit changes in inflammatory response and lung remodeling when subjected to ovalbumin (OVA) sensitization/challenge in adult life. Mice born to dams consuming either HFD or standard chow had increased bronchoalveolar lavage (BAL) levels of IL-1β, IL-4, IL-5, IL-10, IL-13, TNF-α and TGF-β1 after challenge with OVA. IL-4, IL-13, TNF-α and TGF-β1 levels were further increased in the offspring of HFD-fed mothers. Mice born to obese dams also had exacerbated values of leukocyte infiltration in lung parenchyma, eosinophil and neutrophil counts in BAL, mucus overproduction and collagen deposition. The programming induced by maternal obesity was accompanied by increased expression of miR-155 in peripheral-blood mononuclear cells and reduced miR-133b in trachea and lung tissue in adult life. Altogether, the present data support the unprecedented notion that the progeny of obese mice display exacerbated responses to sensitization/challenge with OVA, leading to the intensification of the morphological changes of lung remodeling. Such changes are likely to result from long-lasting changes in miR-155 and miR-133b expression.

## 1. Introduction

The global prevalence of allergic asthma has continuously increased since the last decade of the 20th century [1]. From a clinical perspective, distinct types of asthma are commonly hallmarked by chronic airway inflammation, remodeling of the airway wall and airway hyperresponsiveness. The inflammatory response of allergic asthma is classically recognized as a predominantly T_H_2 activation that leads to IgE production and eosinophil development and infiltration in the lung parenchyma. Such features are granted by T_H_2 cytokines such as interleukin (IL)-4 and IL-5 [2]. IL-13 has also been shown to play a role in airways hyperresponsiveness, mucous secretion and eosinophil recruitment [3,4,5]. More recent is the notion that exacerbation of allergic asthma is also supported by lung neutrophil accumulation, increased production of acute phase proteins, such as the proinflammatory cytokines tumor necrosis factor-α (TNF-α) and IL-1β, and activation of nuclear factor-κB (NF-κB) [6,7].

Along with the history of exposure to the allergen, the incidence of asthma has other determinants. Prospective cohort studies have revealed that increased body mass index (BMI) is significantly related to the risk of asthma in adults [8,9]. A causal relationship between asthma and obesity has been further demonstrated by the improvement of pulmonary function in asthmatic obese patients subjected to weight loss [10]. Obese asthmatic patients have also been described to display exacerbated eosinophilic activation compared to their non-obese counterparts [11]. Accordingly, mice rendered obese by the consumption of a high-fat diet (HFD) and subjected to the model of sensitization and challenge with ovalbumin (OVA) display increased lung eosinophil infiltration and elevated production of both T_H_2 cytokines and acute phase proteins such as IL-6, TNF-α and IL-1β [12,13,14,15].

The impacts of obesity on the intensity and frequency of asthma also have a transgenerational aspect. Human studies have shown that maternal obesity during pregnancy and increased gestational weight gain were positively associated with the risk of childhood asthma [16,17]. In accordance with those findings, it was also described that non-allergen-sensitized mice and rats born to mothers exposed to HFD during pregnancy and lactation spontaneously develop lower respiratory system compliance and higher respiratory system resistance [18,19]. Similar data were found in adult non-allergen-sensitized offspring of mice breastfed by dams consuming HFD [20].

The current study was conducted to evaluate if maternal obesity also affects the inflammatory response in offspring subjected to a model of allergic airway disease. We investigated multiple aspects of eosinophilic inflammation and lung remodeling after sensitization and challenge with OVA in the offspring of mice born to mothers fed HFD. The expression of intracellular signaling proteins and miRNAs associated with the inflammatory response were also evaluated as candidate mechanisms for immune programming.

## 2. Materials and Methods 

### 2.1. Animals and Experimental Design

Four-week-old female C57BL/6J mice were obtained from the Animal Breeding Center at the University of Campinas (CEMIB, Campinas, Sao Paulo, Brazil) and were housed at 22 ± 2 °C under a 12:12 hours light:dark cycle (lights on at 7:00 a.m.) with free access to standard chow (SC) and water for 2 weeks. Two weeks later, female mice were either kept on SC or offered a HFD ad libitum with 22.36 kJ·g^−1^ (60% fat, 15% protein, 25% carbohydrates). Six weeks later, both SC- and HFD-fed mice were mated (housing one male with two females for 3 days). The same diet offered before mating was kept for both groups throughout pregnancy and lactation. The number of pups was adjusted to 5–6 per litter no later than 24 h after delivery. Twenty-one days after delivery, the male offspring of SC- and HFD-fed dams were weaned to the SC diet (originating SC offspring and HFD offspring, respectively).

We used two independent cohorts of pregnant mice in this study. In the first cohort, we generated six litters from HFD-fed mice and six litters of SC-fed mice. Two male littermates per litter belonging to the progenies of both SC- and HFD-fed mothers were sensitized with OVA (cat. no. A5503; Sigma-Aldrich Co; St Louis, MO, USA) by reaching 6 weeks of age. Sensitization consisted of one (i.p.) injection with OVA (30 μg of OVA plus 0.9 mg Al(OH)_3_ diluted in 400 µL of 0.9% NaCl) followed by two additional injections performed 7 and 11 days after the first injection. Three days after the last i.p. injection, one sensitized mouse from each litter was challenged with intranasal installations containing OVA (10 µg/40 µL) (two instillations per day for two consecutive days). Sensitized/challenged mice were thereafter identified as SC/OVA or HFD/OVA. The remaining sensitized littermates were kept unchallenged and received intranasal installations containing vehicle only. These mice were thereafter identified as SC/SHAM or HFD/SHAM. The results showing four different groups were therefore obtained with the mice of the first cohort.

The second cohort was consisted with 12 pregnant HFD-fed mice and 12 pregnant SC-fed mice. One mouse of each litter was allocated in the SC/OVA or in the HFD/OVA subgroup. The remaining littermates were discarded. Therefore, the results showing two different groups were obtained with the mice of the second cohort.

The same weight balance was used throughout the experiments to assess body mass of the dams and of the offspring.

All the experiments were performed 48 h after the beginning of the intranasal challenge, and the procedures were approved by the State University of Campinas Committee for Ethics in Animal Experimentation (protocol no. 3875-1) and were conducted in accordance with the guidelines of the Brazilian College for Animal Experimentation.

### 2.2. Bronchoalveolar Lavage (BAL) Sampling 

The mice were euthanized with sodium thiopental (80 mg/kg), and the trachea was exposed and subsequently cannulated with a polyethylene catheter. The lungs were washed with three flushes containing 500 μL of 0.9% NaCl solution. The volumes were recovered together to produce one BAL sample of approximately 1.5 mL. BAL samples were centrifuged for 10 min at 4 °C (500× *g*) and the supernatants were collected and stored at −80 °C for cytokine determination. The pellets were resuspended in 1.5 mL of 0.9% NaCl solution and used either for flow cytometry or for staining and differential counting of leukocytes as previously described [12].

### 2.3. Flow Cytometry

Aliquots containing 200 µL of BAL samples were centrifuged, and the pellets were suspended in staining buffer (SB) (cat. #554656; BD Biosciences, San Jose, CA, USA) and incubated with Alexa Fluor 647-conjugated anti-mouse CCR3 (cat. #557974; BD Biosciences, San Jose, CA, USA) (0.4 μg per 200 μL) and PE-conjugated anti-mouse VLA4 (cat. #557420; BD Biosciences, San Jose, CA, USA) (0.36 μg per 200 μL) for 20 min at 4 °C. After incubation, the samples were washed three times with SB and fixed (cat. #554655; BD Biosciences, San Jose, CA, USA) for 20 min at RT. After fixation, cells were subjected to an additional washing and reconstituted in SB to a final volume of 300 µL. Samples were added with 50 µL of CountBright beads (54,000 beads/µL) (cat. #C36950; Life Technologies, Carlsbad, CA, USA) for absolute cell counting and then analyzed in a BD FACSCalibur Cytometer (San Jose, CA, USA). Final volume of every sample was 350 µL.

FSC and SSC parameters were acquired in linear scale, while fluorescent signals were acquired in logarithmic scales. Two gates were placed in the FSC versus SSC dot plot. A gate excluding the beads and encompassing the leukocytes was used to evaluate fluorescence (R1). A total of 10,000 events were acquired in R1. The second gate placed in the FSC versus SSC dot plot encompassed the beads population (R2). The absolute number of beads in R2 was used to calculate the volume of each sample that was consumed by the cytometer (consumed volume).

In parallel, we assessed the fluorescent signal of the events acquired in R1 gate. Fluorescent signals were placed in the dot plot of FL2-H (VLA4-positive cells) against FL4-H (CCR3-positive cells). The fluorescence dot plots were divided into four quadrants and the events appearing in the upper right quadrant were considered CCR3^+^/VLA4^+^ events. The absolute number of CCR3^+^/VLA4^+^ events acquired in the consumed volume, the consumed volume and the final volume were used to estimate the whole number CCR3^+^/VLA4^+^ events in each sample. The fluorescent signal obtained from samples stained with Alexa Fluor 647-conjugated (cat. #557690; BD Biosciences, San Jose, CA, USA) and PE-conjugated (cat. #552784; BD Biosciences, San Jose, CA, USA) negative isotypes were previously used to set unspecific fluorescence, and therefore, the dimensions of the lower left quadrant in the fluorescence dot plots. All acquisitions, plots and analyses were obtained using the BD CellQuest Pro software (San Jose, CA, USA).

### 2.4. Cytokine and IgE Determinations

Cytokines were measured in the supernatants of BAL samples using commercially available Enzyme-Linked Immunosorbent Assay (ELISA) kits. The kits for IL-4 (cat. #555232), IL-5 (cat. #555236) and IL-10 (cat. #555252) were from BD Biosciences (San Jose, CA, USA), and the kits for IL-13 (cat. #M1300CB), IL-1β (cat. #DY401), TNF-α (cat. #DY410) and transforming growth factor-β1 (TGF-β1) (cat. #DY1679-05) were from R&D Systems (Minneapolis, MN, USA). IgE levels were determined in plasma samples using ELISA kits (cat. 555248; BD Biosciences, San Jose, CA, USA). Leptin levels were determined in plasma samples using EIA kit (EZML-82K; Merck Millipore, Billerica, MA, USA). Triglycerides, glucose and cholesterol concentrations were determined in plasma samples using colorimetric enzymatic assays (respectively, Cat. No. 1770290, 1770130 and 1770080; LaborLab, Sao Paulo, Brazil).

### 2.5. Lung Histology and Immunohistochemistry

The inferior lobes of the right lungs were excised, immersed in 10% (wt/vol) formalin fixative solution for 72 h and embedded in paraffin. Serial sections (5 μm thick) were mounted onto aminopropyltriethoxysilane-coated glass slides and sequentially processed for paraffin removal with xylol followed by rehydration. Sections were subjected either to different staining protocols or to immunohistochemical detection of phosphorylated NF-κB and tumor necrosis factor alpha-induced protein 3 (TNFAPI3).

Staining with hematoxylin and eosin (H&E) was performed to assess leukocyte infiltrated area. Two random pictures (20× magnification) from each section were analyzed (one random section per animal). The fields were chosen to contain one or two bronchioles. The peribronchiolar leukocyte infiltrated area was manually outlined and its value (excluding the bronchiolar lumen) in µm^2^ was automatically determined with the ImageJ software (http://imagej.nih.gov/ij). To estimate the area of neutrophils and eosinophils within the leukocyte infiltrated area, we used the following approach. The perimeters of the bronchioles were carefully examined using a light microscope equipped with 100× objective lens for use with immersion oil (Nikon Eclipse E200; Nikon, Tokyo, Japan). The percentage of eosinophil and neutrophils in the first layer of leukocytes adjacent to the bronchiolar epithelial cells was estimated. Next, it was assumed that the eosinophil and neutrophil infiltrated areas were a fraction of the leukocyte infiltrated area that were directly proportional to the percentage of eosinophil and neutrophils found in the first layer of leukocytes adjacent to the bronchiolar epithelial cells.

The Masson’s trichrome protocol was performed to determine collagen accumulation. The presence of blue-stained collagen fibers was visualized by optical microscopy under 20× magnification. Images were acquired under 20× magnification using a Leica DM4500 B optical microscope coupled to a digital camera (Leica Microsystems; Wetzlar, Germany) and analyzed with Image-Pro Plus software (version 2.0, Media Cybernetics; Rockville, MD, USA). The blue-stained area with collagen fibers was automatically detected by the software and expressed as the percentage of the tissue section area. The tissue section area was calculated by the software and was defined as the picture area subtracted of the bronchioles and vessels lumen area (empty bronchioles and vessels lumen area was automatically outlined by the software).

The periodic acid-Schiff (PAS) method was performed to determine the mucus content in the epithelial layer. The presence of purple-magenta stained mucus was visualized by optical microscopy under 20× magnification. Images were acquired under 20× magnification using a Leica DM4500 B optical microscope coupled to a digital camera and analyzed with Image-Pro Plus software. The purple-magenta stained area containing mucus was automatically detected by the software and expressed as the percentage of the tissue section area. Calculations were performed as for collagen stained area. Pictures of sections subjected to the PAS method were also used to determine the number of globet cells around the bronchiole perimeter. To this end, the perimeters of the bronchioles were carefully by applying a 2× digital zoom to the images. The number of globet cells (defined as cells containing unstained vacuoles) were manually counted around the bronchiole perimeter and expressed as number of cells per 100 µm.

Sections used for immunohistochemistry were washed with 0.05 M Tris buffered saline (TBS) (pH 7.4) and then incubated with 0.1 M sodium citrate buffer containing 0.05% Tween-20 (pH 6.0) for 24 min at 98 °C for antigen retrieval. Endogenous peroxidase activity was blocked with 0.3% hydrogen peroxide followed by permeabilization with TBS containing 0.1% Tween-20 and 0.5% bovine serum albumin (BSA) at room temperature. Primary antibodies against TNFAIP3 (cat. no. bs-2803R-B; InsightTech; Woburn, MS, USA) or against phospho-NF-κB p65 (S536) (cat. no. ab86299; Abcam; Cambridge, UK) were used at a final dilution of 1:200. Incubations were performed in TBS containing 0.5% BSA overnight at 4 °C. Subsequently, sections were washed with TBS and incubated with biotinylated mouse anti-rabbit IgG, avidin and biotinylated HRP (cat. no. SC-2018; St. Cruz Biotechnology; St. Cruz, CA, USA) following the manufacturer’s instructions. These antibodies have been previously tested in lung sections in parallel to primary omission controls. The area stained for A20 or NF-κB was detected with 3,3′-diaminobenzidine (Sigma Chemical, St Louis, MO, USA) solution. All slides were counterstained with hematoxylin and mounted for observation by microscopy. Images were acquired under a final magnification of 20× using a Leica DM4500 B optical microscope coupled to a digital camera and analyzed with Image-Pro Plus software. The brown-stained areas present both in the sections stained with phospho-NF-κB p65 or in the sections stained with TNFAIP3 were automatically detected by the software and expressed as the percentage of the tissue section area. The tissue section area was calculated by the software and was defined as the picture area subtracted of the bronchioles and vessels lumen area (empty bronchioles and vessels lumen area was automatically outlined by the software).

Two random pictures for each section (one random section per animal) were analyzed in order to originate the data for collagen, mucus, TNFAIP3 and phospho-NF-κB p65 stained area. One bronchiole per picture (two pictures per section, one section per animal) were analyzed to originate the data on globet cell numbers.

### 2.6. RNA Extraction and mRNA and miRNA Detections

A fragment of lung weighing approximately 100 mg, a segment of the trachea and mononuclear cells from whole blood and from spleen were immediately processed for total RNA extraction with Qiazol following the manufacturer’s instructions. PBMCs were obtained from 1 mL of whole blood using Ficoll-Paque PLUS (cat no. 17-1440-02; GE Life Sciences; Uppsala, Sweden) manufacturer’s instructions. For SMCs, whole spleen cells were subjected to hemolysis followed by washing in Dulbecco’s Minimum Essential Medium (DMEM). After washing, the remaining cells were subjected to the Ficoll-Paque method for mononuclear separation exactly as for whole-blood samples.

RNA purity (260/280 and 260/230 ratios) and concentration were calculated using a NanoDrop 2000 spectrophotometer (Thermo Scientific, Waltham, MA, USA) and subjected to poly(A) tailing and reverse transcription with an annealed adaptor (5′-GGCCACGCGTCGACTAGTAC(T)12-3′) as previously described [21]. PCRs were conducted using KAPA SYBR^®^ FAST qPCR Master Mix (Kapa Biosystems, Inc., Boston, MA, USA) in a StepOnePlus Real-Time PCR System (Applied Biosystems, Foster City, CA, USA), using adhesive-sealed plates. The primer sequences were as follows: miRNA antisense (universal): 5′-GGCCACGCGTCGACTAGTAC-3′; miR-133b (miR-133-3p) sense: 5′-TTTGGTCCCCTTCAACCAGCTA-3′; miR-155 (miR-155-5p) sense: 5′-TTAATGCTAATTGTGATAGGGGT-3′; *Rpl37a* (NM_009084) sense: 5′-GTACACTTGCTCCTTCTGTGGC-3′ and antisense: 5′-AGGTGGTGTTGTAGGTCCAGG-3′; *Il4* (NM_021283) sense: 5′-AGCAACGAAGAACACCACAGA-3′ and antisense: 5′-AAGCACCTTGGAAGCCC.

TAC-3′; *Il5* (NM_010558) sense: 5′-TCAAACTGTCCGTGGGGGTA-3′ and antisense: 5′-CTCGCCACACTTCTCTTTTTGG-3′; *Il10* (NM_010548) sense: 5′-AGGCGCTGTCATCGATTTCTC-3′ and antisense: 5′-CTCTTCACCTGCTCCACTGC-3′; *Il13* (NM_008355) sense: 5′-GGCCCCCACTACGGTCT-3′ and antisense: 5′-TCCTCATTAGAAGGGGCCGT-3′. miRNA primer sequences were based on their respective seed sequences; mRNA primers were designed with GeneRunner software. Specificity of the primers were checked by melting profiles and agarose gel electrophoresis of the amplicons. The miRNA and mRNA expression values were normalized using the geometric mean calculated from the reference gene *Rpl37a*. The fold changes were calculated via the 2^−ΔΔCT^ method. Ct values of targets and reference genes were <35, and of the negative control (non-template reactions) were ≥40.

### 2.7. Statistical Analysis

The results are presented as the mean ± standard error of the mean (SEM). D’Agostino and Pearson normality test was used to check for parametric distribution. Comparisons of data from different offspring were made with two-way ANOVA considering (i) type of maternal diet (SC or HFD) and (ii) exposure to challenge with OVA (challenged or unchallenged). Tukey’s multiple comparison test was used as a post-test. Data from SC- and HFD-fed mothers or from parameters assessed exclusively in challenged offspring were compared with Student’s *t*-test (for parametric distribution) or Mann Whitney test (for non-parametric distribution). Pearson or Spearman correlations were used for parametric or non-parametric data, respectively (GraphPad Prism, Version 8.0, San Diego, CA, USA). Results with *p* values that were less than 0.05 were considered significant. The number of independent measures (*n*) indicated throughout the text refers to animals from different progenies.

## 3. Results

### 3.1. Weight and Biochemical Characteristics of Mice Before Mating and Pregnancy Outcomes

We initially assessed biochemical and body weight parameters to demonstrate that our experimental approach was efficient in inducing obesity and its metabolic consequences just before the beginning of pregnancy. Mice fed either SC or HFD were evaluated one day before mating (except for leptin level, which was assessed 21 days after delivery). Our data revealed that body weight, blood glucose and serum triglycerides, cholesterol and leptin were increased in pregnant mice fed a HFD (respectively, 24%, 33%, 38%, 49% and 611% higher than in SC-fed mice; *p* < 0.05) (Table 1).

We found no differences between the numbers of siblings within the progenies born to SC- and HFD-fed dams. The body weight of the offspring born to HFD-fed mice was reduced on the 3rd day and at the end of the 3rd week of life (21% and 17%, respectively, lower than that of the offspring born to SC-fed mice; *p* < 0.05). This difference in body weight was no longer detected at the end of the 8th week of life. Serum triglyceride, cholesterol, leptin and blood glucose levels were also similar between 8-week-old offspring born to HFD- and SC-fed mice (Table 2).

### 3.2. Increased Eosinophil Accumulation in BAL of OVA-Challenged Mice born to HFD-Fed Mice

**Figure 1:**
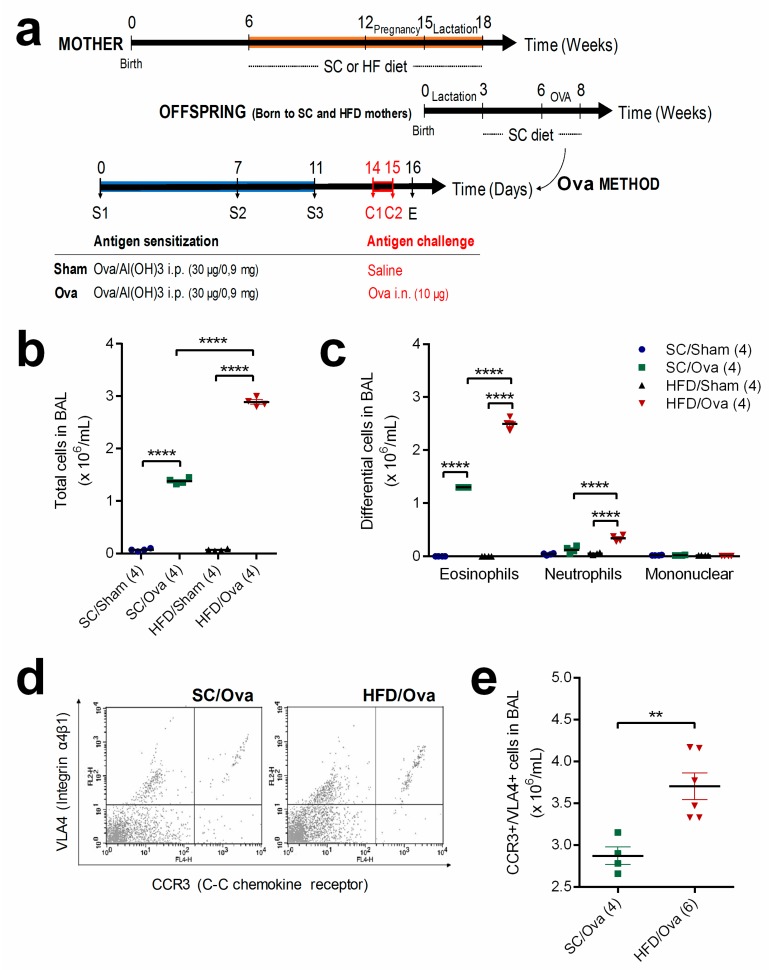
Leukocyte infiltration in the bronchoalveolar lavage (BAL) of mice born to HFD-fed mothers. The offspring born to SC- and HFD-fed mothers were subjected to sensitization only (SHAM) or sensitization and challenge with ovalbumin (OVA) (**a**). BAL samples were obtained from SC/SHAM, SC/OVA, HFD/SHAM and HFD/OVA and processed for total (**b**) and differential leukocyte counting (**c**). BAL samples from SC/OVA and HFD/OVA were also processed for flow cytometry quantification of CCR3^+^/VLA4^+^ cells (**d**,**e**). Data are shown as mean ± SEM. ** *p* < 0.01 and **** *p* < 0.0001.

We conducted multiple measurements to assess if mice born to obese mothers would display an exacerbation of eosinophilic infiltration in BAL and lung parenchyma. A scheme illustrating the protocol for sensitization and challenge with OVA is shown in Figure 1a. Both SC/OVA and HFD/OVA mice had more leukocytes in BAL compared to their unchallenged counterparts (respectively, 20- and 41-fold higher; *p* < 0.0001). However, the number of leukocytes in BAL of HFD/OVA was higher than in that of SC/OVA (2.0-fold; *p* < 0.0001) (*p* < 0.0001 for interaction) (Figure 1b).

Differential counting revealed that eosinophils were only present in the BAL of SC/OVA and HFD/OVA. As observed for total leukocytes, the number of eosinophils in the BAL of HFD/OVA was higher than in that of SC/OVA (1.9-fold; *p* < 0.0001) (*p* < 0.0001 for interaction). Although a prevalence of eosinophils was noted in both challenged groups, challenge with OVA also resulted in an increased number of neutrophils exclusively in BAL of HFD/OVA (6.0-fold higher than in HFD/SHAM and 3.0-fold higher than in SC/OVA; *p* < 0.0001) (*p* = 0.0006 for interaction) (Figure 1c).

Changes in the number of eosinophils in BAL samples were also evaluated by flow cytometry quantification of CCR3^+^/VLA4^+^ cells. In agreement with the results of the differential counting, the flow cytometry experiments revealed that the number of CCR3^+^/VLA4^+^ cells was increased in BAL of HFD/OVA (28% higher than in SC/OVA; *p* = 0.0048) (Figure 1d,e).

**Figure 2:**
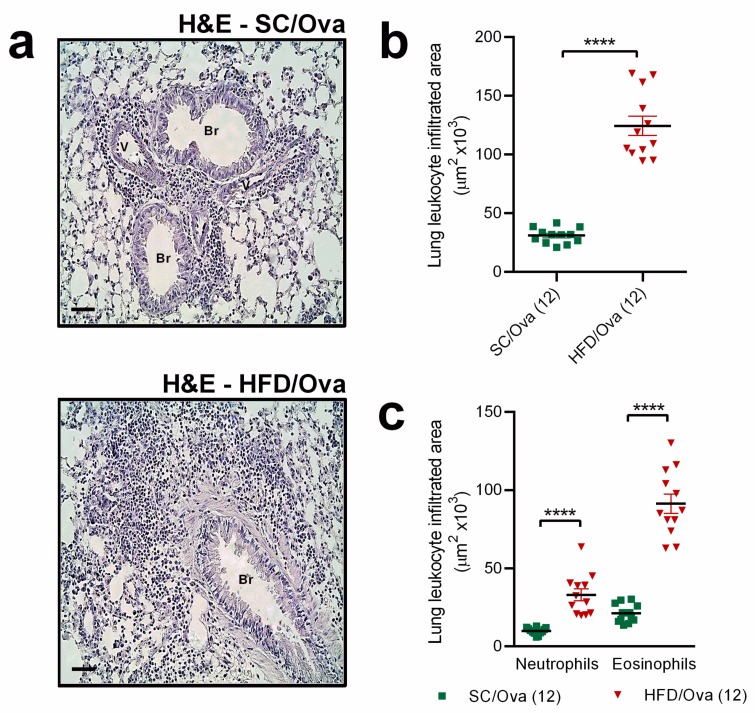
Leukocyte infiltration in the lung parenchyma of mice born to HFD-fed mothers. Lung tissue samples from SC/OVA and HFD/OVA were processed for hematoxylin and eosin (H&E) staining to visualize morphological aspects (**a**). The total leukocyte infiltrated area (**b**) and the neutrophil and eosinophil infiltrated area around the bronchial alveolar space (**c**) were determined using ImageJ software. Horizontal bars represent 50 µm. Data are shown as mean ± SEM. **** *p* < 0.0001. Br: Bronchiole; V: Vessel.

The exacerbation of leukocyte migration to BAL seen in HFD/OVA was paralleled by a more pronounced accumulation of leukocytes in the lung parenchyma, as evidenced by H&E-stained lung sections. H&E staining also revealed that leukocytosis in the peri-bronchoalveolar and peri-vascular spaces, partial destruction of the epithelial layer, smooth muscle hypertrophy and hyperplasia and the development of a subepithelial layer were more pronounced in the HFD/OVA than in the SC/OVA (Figure 2a). The leukocyte infiltrated area in lung parenchyma was increased in HFD/OVA (4.0-fold higher than in SC/OVA; *p* < 0.0001) (Figure 2b). Both the area infiltrated with neutrophils and eosinophils were increased in HFD/OVA (respectively, 3.3- and 4.3-fold higher than SC/OVA; *p* < 0.0001) (Figure 2c).

### 3.3. Increased BAL Levels of Interleukins of the T_H_2 Response and Serum Level of IgE in OVA-Challenged Mice Born to HFD-Fed Mice

**Figure 3:**
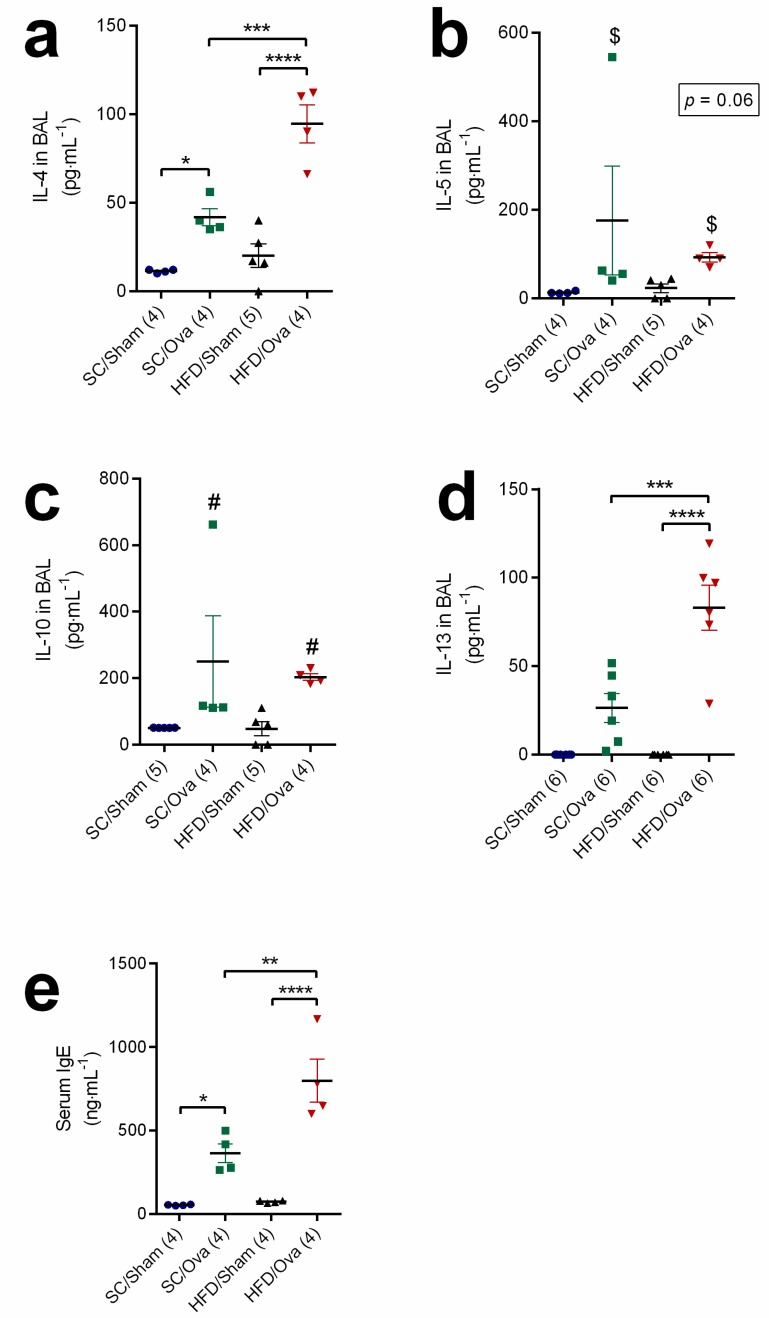
T_H_2 cytokine and IgE levels in mice born to HFD-fed mothers. BAL and serum samples were obtained from SC/SHAM, SC/OVA, HFD/SHAM and HFD/OVA. BAL samples were processed for IL-4 (**a**), IL-5 (**b**), IL-10 (**c**) and IL-13 quantification (**d**). Serum samples were used for IgE determination (**e**). Data are shown as mean ± SEM. * *p* < 0.05; ** *p* < 0.01; *** *p* < 0.001; **** *p* < 0.0001; $ *p* = 0.06 indicating the effect of challenge with OVA and # *p* < 0.05 indicating the effect of challenge with OVA.

We next evaluated IL-4, IL-5, IL-10 and IL-13 BAL levels to elucidate if increased accumulation of eosinophils in BAL and lung of mice born to HFD-fed mice challenged with OVA was associated with a widespread exacerbation of the T_H_2 response. IL-4 level in BAL samples was elevated by challenge with OVA in the SC/OVA and in the HFD/OVA (respectively, 272% and 369% higher than in SC/SHAM and HFD/SHAM; *p* = 0.03 and *p* < 0.0001). IL-4 level in BAL samples of HFD/OVA, however, was higher than in those of SC/OVA (125% higher; *p* = 0.0007) (*p* = 0.0067 for interaction) (Figure 3a).

Although a trend towards an increase in IL-5 in BAL samples was noted in OVA-challenged mice (*p* = 0.06), no specific change was seen when the comparison was made between SC/OVA and HFD/OVA (*p* = 0.43 for interaction) (Figure 3b). Similarly, challenge with OVA equally increased IL-10 in BAL samples from SC/OVA and HFD/OVA (36% higher than in unchallenged mice; *p* = 0.01). However, no specific changes were found when comparing the IL-10 levels of SC/OVA and HFD/OVA (*p* = 0.72 for interaction) (Figure 3c). IL-13 was only detected in BAL samples of challenged mice, but its level in HFD/OVA was higher than in SC/OVA (214% higher; *p* = 0.0005) (*p* = 0.0032 for interaction) (Figure 3d).

As with the BAL level of IL-4, the serum level of IgE was elevated by challenge with OVA in the SC/OVA and in the HFD/OVA (respectively, 575% and 981% higher than in SC/SHAM and HFD/SHAM; *p* = 0.03 and *p* < 0.0001). However, the IgE level of HFD/OVA was higher than in SC/OVA (119% higher; *p* = 0.004) (*p* = 0.012 for interaction) (Figure 3e).

### 3.4. Intensification of Lung Remodeling in OVA-Challenged Mice Born to HFD-Fed Mice

**Figure 4:**
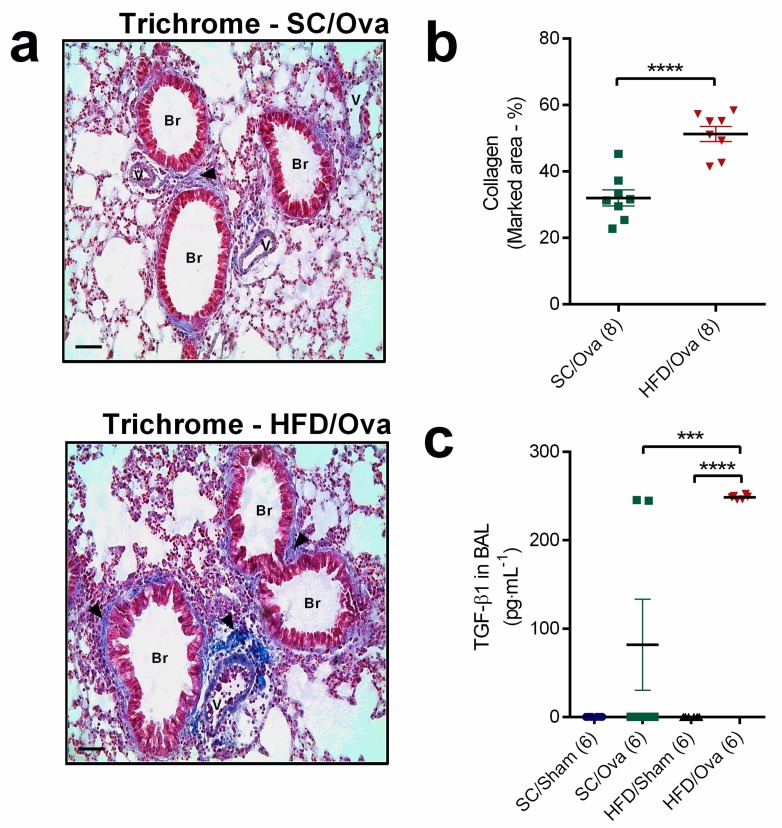
Lung collagen deposition and TGF-β1 level in mice born to HFD-fed mothers. Lung tissue samples from SC/OVA and HFD/OVA were subjected to the Masson’s trichrome staining protocol to assess collagen deposition. Representative images show peri-bronchoalveolar blue-stained collagen fibers (black arrows) (**a**). The percentage of stained area was assessed with Image-Pro Plus software (**b**). BAL samples were obtained from SC/SHAM, SC/OVA, HFD/SHAM and HFD/OVA mice and processed for TGF-β1 quantification (**c**). Horizontal bars represent 50 µm. Data are shown as mean ± SEM. *** *p* < 0.001 and **** *p* < 0.0001. Br: Bronchiole; V: Vessel.

We next evaluated if the overproduction of some T_H_2 interleukins induced by challenge with OVA in mice born to HFD-fed mice could impact the changes associated with lung remodeling in the allergic asthma. Masson’s trichrome staining revealed that both SC/OVA and HFD/OVA had evident collagen deposition in the peri-bronchoalveolar space that was partially located over the subendothelial smooth muscle layer. The percentage of the area stained for collagen was, however, 60% higher in HFD/OVA than in SC/OVA (*p* < 0.0001) (Figure 4a,b). TGF-β1 was not detected in BAL samples of unchallenged mice. On the other hand, TGF-β1 level in BAL samples of HFD/OVA was 255% higher than in those of SC/OVA (*p* < 0.001) (*p* = 0.004 for interaction) (Figure 4c).

**Figure 5:**
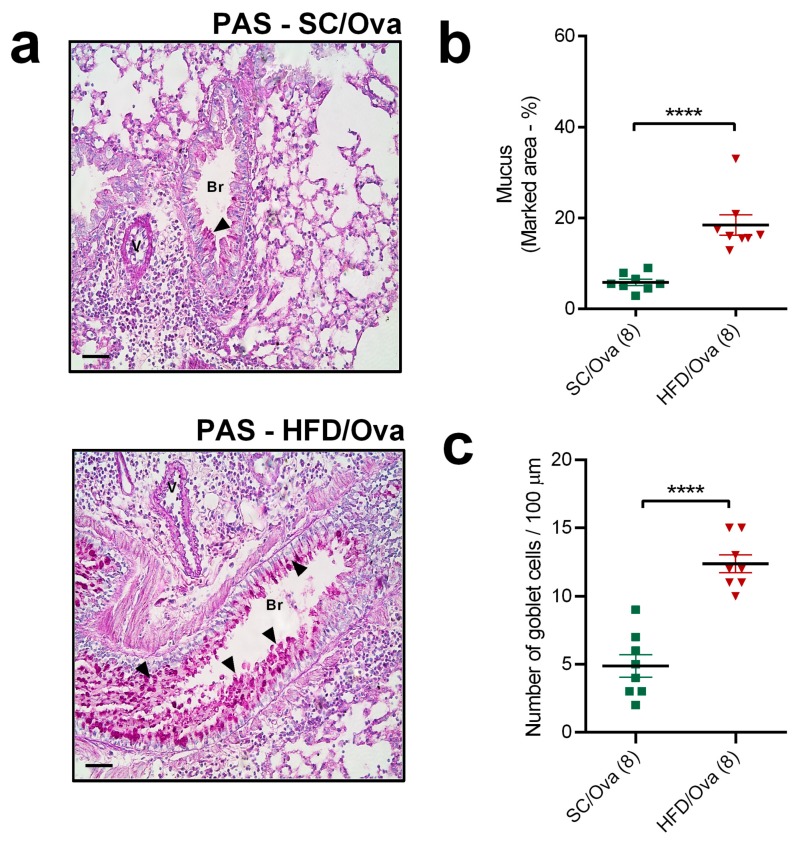
Mucus production in mice born to HFD-fed mothers. Lung tissue samples from SC/OVA and HFD/OVA were subjected to the PAS staining method to assess mucus production. Representative images show purple-magenta-stained mucus over the epithelial layer (black arrows) (**a**). The percentage of stained area was assessed with Image-Pro Plus software (**b**). The number of goblet cells in the broncho epithelial layer was determined by morphological criteria (**c**). Horizontal bars represent 50 μm. Data are shown as mean ± SEM. **** *p* < 0.0001. Br: Bronchiole; V: Vessel.

The PAS method also revealed that both groups of mice challenged with OVA presented purple-magenta staining for mucus over the epithelial layer. This indication of mucus production was exacerbated in HFD/OVA, as evidenced by the quantification of the percentage of purple-magenta-stained area (214% higher than in SC/OVA; *p* < 0.0001) (Figure 5a,b). The number of globet cells in the bronchioles was also increased in HFD/OVA mice (150% higher than in those of SC/OVA; *p* < 0.0001) (Figure 5c).

### 3.5. Mice Born to HFD-Fed Mothers Have Exacerbated TNF-α level and Increased TNF-α Signaling after Being Challenged with OVA

**Figure 6:**
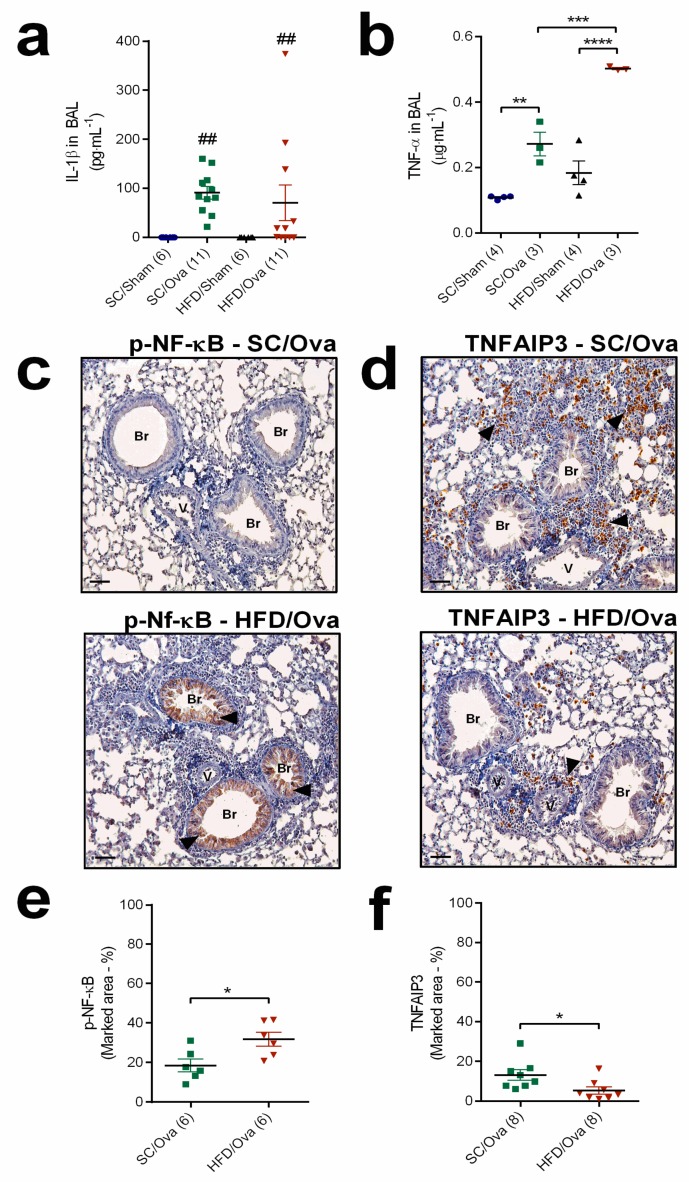
Acute phase protein levels and TNF-α signaling in mice born to HFD-fed mothers. BAL samples were obtained from SC/SHAM, SC/OVA, HFD/SHAM and HFD/OVA mice and processed for IL-1β (**a**) and TNF-α quantification (**b**). Lung tissue samples from SC/OVA and HFD/OVA were subjected to immunohistochemistry detection of phospho-NF-κB (**c**) and TNFAIP3 (**d**) (black arrows). The percentages of phospho-NF-κB- (**e**) and TNFAIP3-stained areas (**f**) were assessed with Image-Pro Plus software. Horizontal bars represent 50 µm. Data are shown as mean ± SEM. * *p* < 0.05; ** *p* < 0.01; *** *p* < 0.001; **** *p* < 0.0001 and ## *p* < 0.01 indicating the effect of challenge with OVA. Br: Bronchiole; V: Vessel.

To evaluate if the exacerbation of the inflammatory response to allergic asthma in mice born to HFD-fed mothers was not restricted to the T_H_2/eosinophil axis, we also assessed the production of proinflammatory cytokines that act as acute phase proteins. BAL IL-1β was only detected in samples of OVA-challenged mice, but no difference was noted between HFD/OVA and SC/OVA (*p* = 0.72 for interaction) (Figure 6a). TNF-α concentration was also elevated in BAL samples of SC/OVA and HFD/OVA mice (respectively, 172% and 177% higher than in SC/SHAM and HFD/SHAM; *p* = 0.006 and *p* < 0.0001). In contrast to IL-1β, the level of TNF-α in BAL of HFD/OVA was higher than in those of SC/OVA (86% higher; *p* = 0.0008) (*p* = 0.01 for interaction) (Figure 6b).

We also evaluated two intracellular proteins that take part in the cellular signaling evoked by TNF-α. The area stained for phosphorylated NF-κB was higher in lung sections of HFD/OVA than in SC/OVA (71% higher; *p* = 0.02) (Figure 6c,e). On the other hand, the area stained for TNFAIP3, a repressor of TNF-α signaling, was reduced in lung sections of HFD/OVA (60% lower than in SC/OVA; *p* = 0.03) (Figure 6d,f).

### 3.6. Mice Born to HFD-Fed Mice Have Increased miRNAs That Are Associated with the Exacerbation of the T_H_2 Response and IL-13 Signaling

The present investigation also sought to clarify the mechanisms underlying the exacerbated allergic airway inflammation detected later in the life of the progeny born to obese mothers. We chose to study miR-133b based on the following reasons. A study profiling several circulating miRNAs in serum samples has shown that miR-133b was one miRNA that was downregulated in asthmatic patients [22]. Evidence showing that miR-133b is more than only a biomarker for allergic asthma came from studies with mice demonstrating that the model of sensitization and challenge with OVA leads to a reduction in miR-133b expression in nasal mucosa. It was further demonstrated that upregulation of miR-133b with an specific agomir was able to abrogate OVA-induced increased in IgE, IL-4, IL-5 and TNF-α levels and lung eosinophil infiltration [23].

Investigating the expression of miR-133b in our model was also of particular interest because this microRNA was described to modulate the proliferation of smooth muscle cells in different territories. Increased expression of miR-133b was associated to reduced proliferation and calcium levels in vascular smooth muscle cells [24,25]. Additionally, it was also shown that TGF-β1-induced reduction of miR-133b expression in bladder smooth muscle cells was pivotal for collagen accumulation in these cells [26].

Due to the reasons stated above, and based on our data on collagen deposition and TGF-β1 levels in BAL, we decided to evaluate miR-133b expression in the lung and in the trachea of HFD/OVA and SC/OVA mice.

The choice for the study of miR-155 was based on consistent literature showing its participation in the T_H_2 response of allergic airway disease. Mice knockout for miR-155 exhibited attenuated eosinophil infiltration in the lung parenchyma, reduced mucus production and lower IL-4, IL-5 and IL-13 levels when subjected to OVA sensitization and challenge [27]. Concordant data using mice knockout for miR-155 were found by other groups [28,29]. Accordingly, miR-155 overexpression resulted in increased mucus production, lung eosinophil infiltration and IL-4, IL-5 and IL-13 levels [30]. Directly inhibiting miR-155 expression in T_H_2 cells prevents T_H_2-mediated airway allergy (airway eosinophilia, IL-13 production and airway mucus production [31]). miR-155 expression was also described to be positively associated in IL-4, IL-5 and IL-13 production by CD4^+^ cells isolated from allergic patients [32]. On the other hand, miR-155 was reported to reduce IL-13-induced bronchial smooth muscle cells proliferation and migration [33]. Therefore, we decided to evaluate miR-155 expression not only in peripheral-blood mononuclear cells (PBMCs) and in splenic mononuclear cells (SMC), but also in lung and trachea of HFD/OVA and SC/OVA mice.

**Figure 7:**
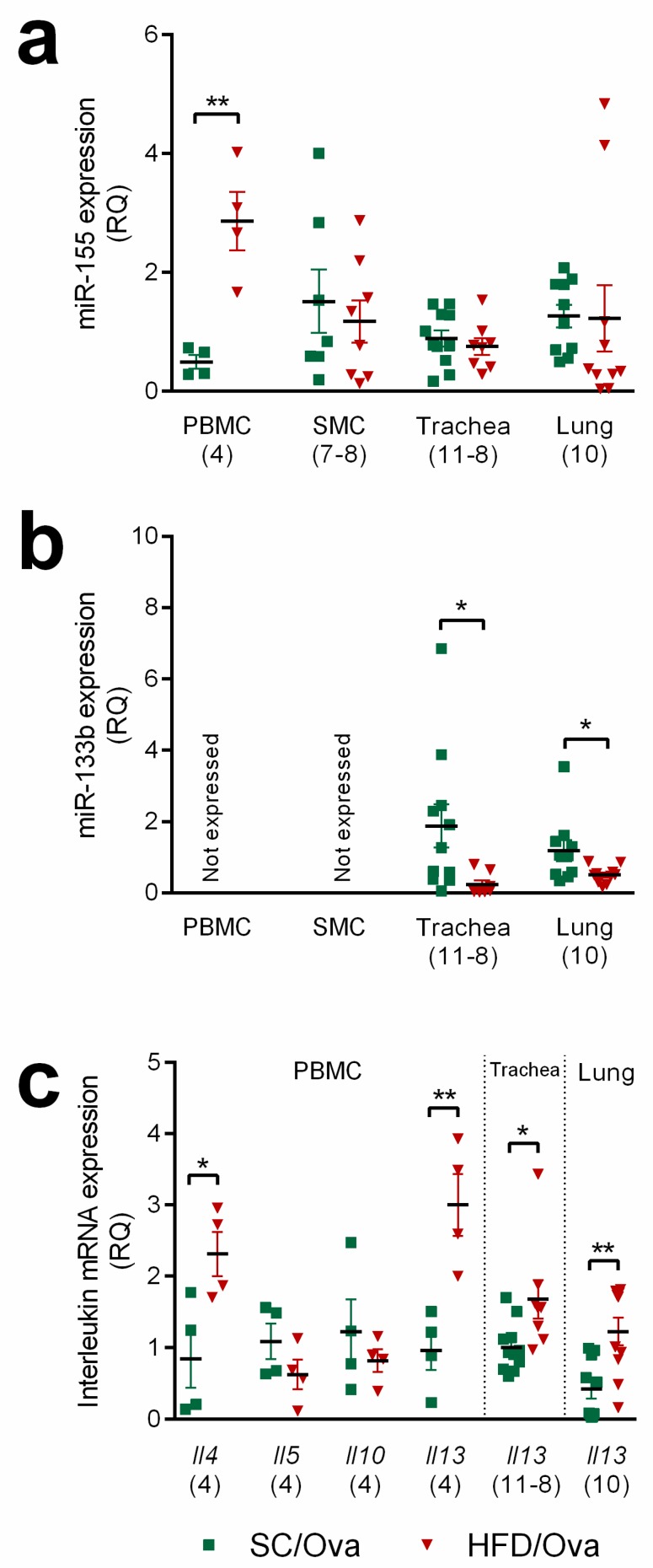
miR-155, miR-133b and interleukins expression in mice born to HFD-fed mothers. PBMC, SMC, trachea and lung tissue samples were obtained from SC/OVA and HFD/OVA and processed for RNA extraction and miR-155 (**a**) and miR-133b detection (**b**) by Quantitative polymerase chain reaction (qPCR). Expression of interleukins *Il4*, *Il5*, *Il10* and *Il13* mRNA in PBMC, and *Il13* mRNA in trachea and lung were also determined. (**c**) *Rpl37*a was used as an internal control. Data are shown as mean ± SEM. * *p* < 0.05 and ** *p* < 0.01.

The expression of miR-155 was upregulated in PBMCs of HFD/OVA mice (483% higher than in SC/OVA; *p* = 0.003). No significant changes were found in SMC or trachea or lung samples (Figure 7a). The expression of miR-133b was not detected in PBMCs or in SMCs, but its level was reduced in the trachea and in the lung samples of the HFD/OVA (respectively, 87% and 58% lower than in SC/OVA; *p* = 0.03) (Figure 7b).

In order to explore the relevance for the increased miR-155 in PBMC of HFD/OVA mice, we evaluated the mRNA expression of *Il4*, *Il5*, *Il10* and *Il13*. In agreement with our data on interleukin concentration in BAL, we found that HFD/OVA mice had increased *Il4* and *Il13* expressions in PBMC (respectively, 175% and 212% higher than in SC/OVA; *p* = 0.027 and *p* = 0.007). No changes were found in *Il5* and *Il10* expression when comparing PBMC samples of HFD/OVA and SC/OVA (Figure 7c).

The relevance for the reduced miR-133b in trachea and lung samples of HFD/OVA mice was explored by measuring the *Il13* mRNA expression in these samples. The expression of *Il13* was increased both in trachea and lung samples of HFD/OVA (respectively, 67% and 190% higher than in SC/OVA; *p* = 0.019 and *p* = 0.003) (Figure 7c).

**Figure 8:**
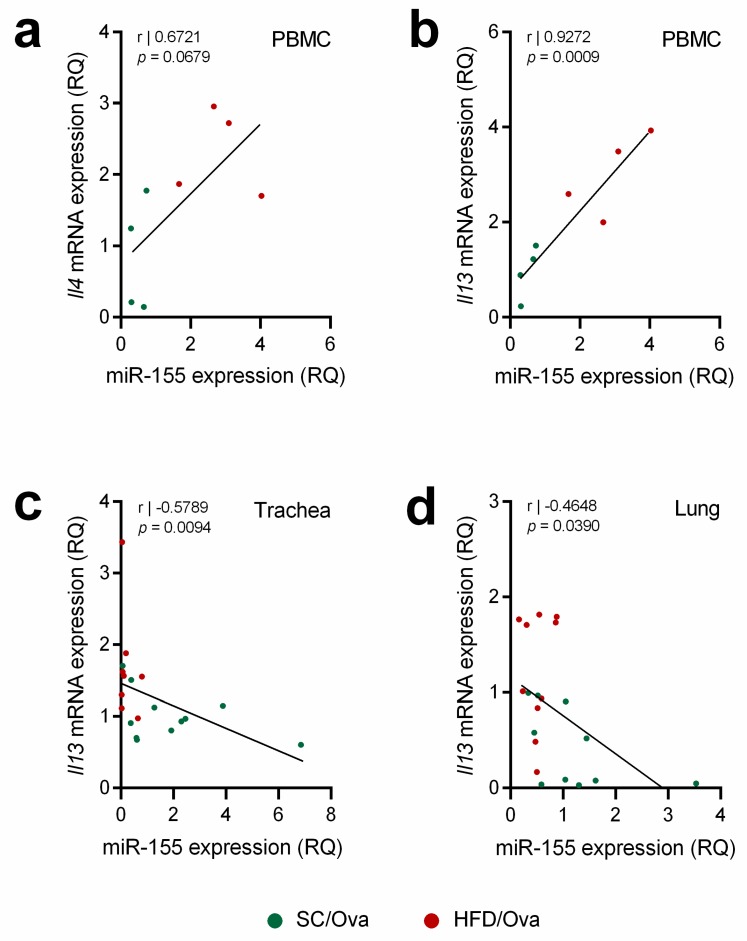
Correlation of interleukin expression with miRNAs in mice subjected to sensitization and challenge with OVA. The expression miR-155 in PBMC of SC/OVA and HFD/OVA was correlated with *Il4* (**a**) and *Il13* (**b**) expressions. The expression miR-133b in trachea (**c**) and in lung (**d**) of SC/OVA and HFD/OVA was correlated with *Il13* expression.

We also tested whether the levels of miR-155 and miR-133b would correlate with the levels *Il4* and *Il13* expressions. We detected a trend towards a positive correlation between miR-155 and *Il4* expressions in PBMC (*r* = 0.6721; *p* = 0.0679) and a positive correlation between miR-155 and *Il13* expressions in PBMC (*r* = 0.9272; *p* = 0.0009) (Figure 8a,b). On the other hand, a negative correlation between miR-133b and *Il13* expressions were found in trachea (*r* = −0.5789; *p* = 0.0094) and lung samples (*r* = −0.4648; *p* = 0.039) (Figure 8c,d).

## 4. Discussion

Recent observational studies have shown that children born to obese women are more prone to develop asthma [16,17]. The increased risk for asthma in children born to obese mothers was observed using models that were adjusted for several co-varieties including mother’s race, maternal age and child’s sex [34]. Apart from these studies, no clear mechanism for this relationship has been suggested thus far. To the best of our knowledge, this is the first study to demonstrate that mice born to and breastfed by obese mothers exhibit exacerbated activation of inflammatory parameters when subjected to the model of allergic airway inflammation characterized by sensitization followed by challenge with OVA.

The present data are in accordance with previously published papers showing persistent respiratory abnormalities in offspring born to HFD-fed dams. Using OVA-naive offspring, a previous report by Griffiths et al. demonstrated that rats born to mothers fed a HFD during pregnancy and lactation display increased levels of acute phase proteins, such as IL-1α, IL-1β and TNF-α, along with an increase in the T_H_2 cytokine IL-5, during the first days of life. Some changes were persistently observed at the moment of weaning and in the adult offspring. Those authors have also demonstrated that weanling rats born to and breastfed by HFD-fed dams had higher basal respiratory resistance and lower respiratory compliance, although some of these changes disappeared when the progeny reached adult life [19].

MacDonald et al. have demonstrated that the male and female offspring of BALB/cByJ mice born to and breastfed by mothers receiving HFD manifest increased methacholine-induced respiratory resistance and increased neutrophil count and IL-6 level in BAL samples [18]. Dinger et al., instead, used C57B6 mice to show that feeding dams with HFD during lactation caused greater airway resistance in the male offspring not exposed to OVA [20]. Given the data published by Dinger et al. [20], we decided to carry our experiments in the male offspring of C57BL/6J mice.

The present study further contributes to this field of investigation by demonstrating that sensitization followed by challenge with OVA leads to an exacerbated increase in IL-4, IL-13 and TNF-α in BAL of male C57BL/6J mice born to and breastfed by HFD-fed dams. Such findings support the notion that changes imprinted on the immune system may play an important role in the respiratory programming induced by maternal obesity.

We have found no differences in leukocytes in BAL when comparing SC/SHAM to HFD/SHAM mice. This is in apparent contrast to the study published by MacDonald et al. [18]. Those researchers found increased leukocytes in BAL of mice born to HFD-fed mice. However, there are two important differences between the study by MacDonald et al. and the present experiments that may contribute such different results. The offspring in the study by MacDonald et al. were naive to OVA while our SHAM animals were sensitized with the allergen. The study by MacDonald et al. used a strain of BALB/c mice, while we used C57Bl/6J mice.

Interestingly, our data show that, in contrast to IL-4 and IL-13, challenge with OVA did not promote a further increase in IL-10 and IL-5 levels in BAL of mice born to HFD-fed dams. Such selective modulation demonstrates that the programming induced by maternal obesity is not characterized by a widespread activation of the T_H_2 response in the offspring. On the other hand, it is noteworthy that published evidence clearly supports the proposition that overproduction of IL-4 and IL-13 may be responsible for the features of allergic asthma that are exacerbated in the offspring born to HFD-fed mice after challenge with OVA.

IL-4 is known to act in uncommitted B cells, promoting their switch to an IgG1- and IgE-secreting phenotype [35]. Therefore, an exacerbated IL-4 level in BAL samples explains the increased serum IgE found in HFD/OVA mice. Moreover, IL-4 has been shown to boost the airway eosinophilia induced by IL-5 in mice sensitized/challenged with OVA [36]. Accordingly, we found increased eosinophils in the lung parenchyma and in the BAL of HFD/OVA mice. Evidence also exists supporting the proposition that exacerbated IL-13 level in BAL of OVA-challenged mice born to HFD-fed dams may contribute to their increased lung eosinophilia [3].

IL-10, instead, is produced by Treg cells and exerts regulatory function of T_H_1 and T_H_2 cells [37]. It was already described that IL-10 production by thymic Foxp3-negative Treg cells alleviates the lung eosinophil infiltration, globet cell hyperplasia and IgE and production in mice subjected to the OVA model of allergic asthma [38]. Our data revealed that the male offspring born to either HFD-fed or SC-fed mothers exhibit a similar increase in BAL IL-10 levels when challenged with OVA. Thus, we can conclude that the exacerbated features of allergic airway inflammation seen in the offspring born to HFD-fed mothers are probably not due to an impaired IL-10 production by Treg cells.

The exacerbated presence of eosinophils in BAL of OVA-challenged mice born to HFD-fed dams was also evidenced by the increased concentration of CCR3^+^/VLA4^+^ cells. CCR3, also known as CD193, was described to play a pivotal role in the transendothelial migration of activated eosinophils [39]. VLA4, also known as α4β1 integrin, is highly expressed in eosinophils of asthmatic individuals and contributes to their increased adhesion to airway smooth muscle [40].

Another features induced by challenge with OVA that were intensified in mice born to obese mothers were mucus hypersecretion and globet cells hyperplasia. As we understand, it is reasonable to assume that excessive IL-4 and IL-13 may account for mucus hypersecretion in mice born to HFD-fed mice. Accordingly, previous studies have demonstrated that IL-13 and IL-4 can stimulate mucus production in vivo and in vitro by acting directly in goblet cells [5,41,42,43] stimulating the expression of different mucins such as Muc2 and Muc5-ac [44,45]. IL-4, in turn, was also described to induce structural changes, such as epithelial hypertrophy of the airways, a feature also noted in the offspring born to obese dams [46].

The present study also reveals that exacerbation of the inflammatory response induced by OVA in the offspring born to obese dams has consequences for lung remodeling, as evidenced by increased collagen deposition and BAL TGF-β1 level. Interestingly, TGF-β1 produced by airway smooth muscle cells was described to act in an autocrine fashion to stimulate collagen expression [47,48].

Another aspect revealed by the present study is that OVA-challenged mice born to obese mothers manifested exacerbated TNF-α level along with neutrophils in BAL and increased NF-κB phosphorylation in lung. We also found reduced TNFAIP3 level in the lung of HFD/OVA mice. TNFAIP3 is an inhibitor of NF-κB that was reported to reduce airway leukocyte recruitment, peribronchoalveolar inflammation and mucus production and airway hyperresponsiveness (AHR) in mice subjected to OVA sensitization/challenge [49,50]. Interestingly, TNF-α, by activating NF-κB, was described to play a role in the steroid-resistant neutrophilic inflammation and AHR that occur in chronically sensitized (four weeks) mice subjected to a single moderate OVA challenge [6]. Furthermore, neutrophils and TNF-α levels in the lung were implicated in NF-κB activation in distinct models of lung injury [51,52]. Accordingly, as our data with BAL samples were obtained by washing the entire lungs, it is plausible to assume that, even assessing one random section per animal, our immunohistochemistry data represent phenomena that are taking place in the whole lung.

Our data showing reduced miR-133b in the trachea and in the lung and increased miR-155 in the PBMC indicates a putative mechanism by which the exacerbated response to OVA develops in the adult progeny. Several lines of evidence indicate that miR-133b is relevant to the inflammatory response and lung remodeling in allergic asthma. Lower levels of circulating miR-133b have been detected in asthmatic patients [22], and its overexpression in nasal mucosa was shown to target the Nlrp3 inflammasome and reduce eosinophil infiltration and IgE, TNF-α, IL-4, IL-5 and IFN-γ levels in mice sensitized/challenged with OVA [23].

The reduction in miR-133b was also shown to mediate TGF-β1-induced collagen accumulation in bladder smooth muscle cells [26]. Notably, IL-13-induced collagen synthesis was reported to be dependent on TGF-β1 production [53]. In vivo IL-13 neutralization also resulted in lower collagen deposition in lung of mice subjected to experimental asthma [54]. Our data showing a negative correlation of IL-13 with miR-133b in trachea and lung of the mice belonging to the OVA subgroups further suggest that the downregulation of miR-133b may play a role in the increased collagen deposition seen in the HFD/OVA group.

As we understand, the increased expression of miR-155 in PBMCs found in the offspring born to obese mothers also helps to explain their increased IL-4 and IL-13 levels. It has been demonstrated that allergen stimulation of CD4^+^ cells in vitro increases miR-155 expression and IL-13 production [32]. miR-155-knockout mice subjected to OVA sensitization/challenge showed reduced levels of IL-4, IL-5 and IL-13 as well as lower lung eosinophil/neutrophil infiltration compared to wild-type [27]. Accordingly, miR-155 was described to stimulate the T_H_2 response and mucus hypersecretion by directly targeting sphingosine-1-phosphate receptor 1 and cytotoxic T lymphocyte–associated antigen 4 in CD4^+^ cells in experimental models of asthma [30,31]. The positive correlation between miR-155 with IL-13 and a trend towards a significant correlation with IL-4 in PBMCs of the mice belonging to the OVA subgroups favors the interpretation that elevated miR-155 may be relevant for the increased levels of IL-4 and IL-13 in HFD/OVA.

It is still challenging to determine the mechanism by which maternal obesity increases the intensity allergic outcomes in the offspring. It is now clear that changes in gut microbiota play a crucial role in metabolic adaptations to obesity [55]. Feeding mice with a HFD has been show to induce changes in gut microbiota in parallel to a reduction in fecal content of short chain fatty acids (SCFAs) [56]. SCFAs are produced by the intestinal microbiota fermentation of indigestible dietary carbohydrates such dietary fiber. It has been shown that treatment with several SCFAs including acetate is capable of preventing HFD-induced metabolic outcomes [57]. In this context, a recent study in mice has suggested that diet-induced changes gut microbial diversity in the mother may play a crucial role in the programming of allergic asthma in the offspring. Exposure of pregnant mice to a high fiber diet or to the SCFA acetate in the drinking water resulted in an attenuation of allergic airway disease in the offspring subjected to sensitization/challenge with house dust mite challenge [58].

In summary, our study reveals unprecedented data showing that progeny born to obese mice display exacerbated responses to sensitization/challenge with OVA. This exacerbation is hallmarked by overproduction of IL-4, IL-13, TNF-α and TGF-β1, which is paralleled by increased circulating IgE. Mice born to obese mothers also display accentuated eosinophil/neutrophil infiltration in the lung parenchyma, increased collagen deposition and increased mucus hypersecretion. From the mechanistic point of view, our data suggest that increased miR-155 in PBMCs and reduced miR-133b in lung tissue and trachea are likely to mediate the programming events described herein.

## Figures and Tables

**Table 1:** Body weight and biochemical characteristics of Standard chow (SC) and High Fat Diet (HFD) fed dams.

	SC Dams	HFD Dams	*P*
**N**	12	12	-
**Body mass (g)**	21.68 ± 1.24	27.03 ± 3.89	***
**Triglycerides (mg/dL)**	114.3 ± 27.4	158.1 ± 36.6	**
**Glucose (mg/dL)**	128.8 ± 14.9	170.5 ± 15.54	****
**Cholesterol (mg/dL)**	94.15 ± 7.9	141.2 ± 32.9	***
**Leptin (ng/mL)**	1.555 ± 0.297	11.070 ± 2.130	***
**Number of pups per litter**	6.1 ± 1.6	5 ± 1.9	nd

Body mass, blood glucose and serum triglycerides and cholesterol were assessed 1 day before mating. Serum leptin was evaluated 21 days after delivery. The number of pups per litter was evaluated during the first 24 h after delivery. Data are shown as mean ± SEM. ** *p* < 0.01 versus SC dams; *** *p* < 0.001 versus SC dams; **** *p* < 0.0001 versus SC dams. nd: Not detected.

**Table 2:** Body weight and biochemical characteristics of the offspring born to SC- and HFD-fed dams.

	SC Offspring	HFD Offspring	*P*
**N**	8	8	-
**Triglycerides (mg/dL)**	82.02 ± 29.5	67.16 ± 24.87	nd
**Glucose (mg/dL)**	150.4 ± 24.29	142.4 ± 18.28	nd
**Cholesterol (mg/dL)**	78.36 ± 12.24	67.07 ± 12.08	nd
**Leptin (ng/mL)**	0.588 ± 0.162	0.587 ± 0.064	nd
**Body mass at the 3rd day of life (g)**	3.17 ± 0.14	2.52 ± 0.14	**
**Body mass at the 21st day of life (g)**	9.36 ± 0.61	7.56 ± 0.19	*
**Body mass at the 8th week of life (g)**	26.13 ± 2.34	24.38 ± 0.73	nd

Blood glucose and serum triglycerides, cholesterol and leptin were assessed at the 8th week of life. Data are shown as mean ± SEM. * *p* < 0.05 versus SC offspring; ** *p* < 0.01 versus SC offspring. nd: Not detected.

## Data Availability

Data generated during the study are available from the corresponding author upon reasonable request.
